# Using Point-of-care Ultrasonography to Diagnose Traumatic Arthrotomy of the Knee: A Case Series

**DOI:** 10.5811/cpcem.1919

**Published:** 2024-02-13

**Authors:** Jordan Mullings, Henry Ashworth, Matthew Kongkatong, Daniel Mantuani

**Affiliations:** * Alameda Health System, Department of Emergency Medicine, Highland Hospital, Oakland, California; † University of Virginia Health System, Department of Emergency Medicine, Charlottesville, Virginia

**Keywords:** *case report*, *POCUS*, *traumatic arthrotomy*, *intra-articular air*, *musculoskeletal ultrasound*

## Abstract

**Introduction:**

Accurate diagnosis of traumatic arthrotomy of the knee (TAK) is critical for patients presenting to the emergency department (ED) to ensure timely treatment. Current diagnostic modalities including plain radiography, computed tomography (CT), and the saline load test (SLT) have advantages and disadvantages. Point-of-care-ultrasonography (POCUS) offers a possible timely, low-cost, and efficient alternative method of diagnosing TAK. In this case series we present three cases where POCUS was used to diagnose TAK in the ED.

**Case Series:**

Three patients in their early 20s presented to the ED complaining of knee trauma with wounds in proximity to the joint. Mechanisms of injury included a gunshot wound in one case and blunt trauma (motor vehicle collision and bicycle crash) in two cases. In all three cases TAK was suggested on POCUS examinations by the presence of intra-articular hyperechoic foci consistent with air artifact. All three cases had TAK confirmed by orthopedic evaluation.

**Discussion:**

Ultrasound may have utility in the evaluation of patients presenting with knee trauma where TAK is a concern. The SLT is generally considered the gold standard test for diagnosis of TAK, but it is invasive and has a wide range of diagnostic accuracy. Intra-articular air has been found to be a sensitive marker for TAK in CT studies. Thus, additional investigations into the diagnostic accuracy of POCUS for this finding should be undertaken.

Population Health Research Capsule What do we already know about this clinical entity?

*Traumatic knee arthrotomies can lead to septic arthritis if not identified and treated appropriately.*

What makes this presentation of disease reportable?

*To our knowledge, this is only the second case series where point-of-care-ultrasound (POCUS) was used to accurately diagnose traumatic arthrotomies in living patients.*

What is the major learning point?

*Intra-articular air is readily seen on POCUS of knee joints and has precedent of being a good marker for arthrotomy in other modalities.*

How might this improve emergency medicine practice?

*Identification of intra-articular air on POCUS of joints may allow for the rapid, noninvasive diagnosis of traumatic arthrotomy.*

## INTRODUCTION

Traumatic arthrotomy of the knee (TAK) involves damage to the capsule or supporting structures (eg, ligaments) that results in violation of the joint space.^1^ Typically, traumatic arthrotomies are treated by operative irrigation and debridement, although there is emerging evidence that small, noncontaminated defects can be safely treated at the bedside.^2^ Delay in diagnosis and treatment can result in the development of septic arthritis resulting in significant increases in morbidity (50%) and mortality (11–15%).^3^

Previous studies have documented the diagnostic modalities that are most effective at detecting TAK in cases where it is not obvious on physical examination.^4^ These include a saline load test (SLT) with or without methylene blue and computed tomography (CT). Radiographs are commonly obtained in the evaluation of joint injuries. They have reasonable sensitivity (78%) and high specificity (90%) for TAK when intra-articular air is detected. However, this finding may be subtle and overlooked if the reader is concentrating on evaluation for bony injury.^1^

The SLT as a diagnostic tool can be unpredictable, with sensitivities reliant on multiple factors.^5^ While an increased amount of injected saline leads to improved sensitivity (99% for 175 milliliters (mL), results vary based on joint location, patient tolerance of injection, and time spent on procedure.^1^^,^^5^ Passive ranging of the joint while performing the SLT has also shown some improvements in the sensitivity of the test; however, combining the SLT with methylene blue injections has shown little to no benefit.^5^ There are also some studies reporting that operator proficiency can lead to false negative and false positive results.^5^^,^^6^ The SLT is also painful, particularly when large volumes are used, which may limit the operator’s ability to perform the test thoroughly.

Computed tomography has been investigated as an alternative test for traumatic arthrotomy in recent years. The modality has been shown to be both highly sensitive (100%) and specific for TAK in a cohort of 62 emergency department (ED) patients presenting with wounds around the knee.^7^ In these protocols intra-articular air is used to make the diagnosis and CT is sensitive enough to detect as little as 0.1 mL of air.^8^ Computed tomography has been shown to have similar performance in the evaluation of traumatic arthrotomy of other joints as well.^1^^,^^9^ Computed tomography can also better characterize fracture patterns and inform treatment decisions compared to radiograph.^10^ However, high utilization of CT exposes patients to increased amounts of ionizing radiation, incurs significant cost to the healthcare system, and requires that the patient leave the treatment area.

Since intra-articular air appears to be a useful imaging finding to diagnose TAK, point-of-care-ultrasonography (POCUS) may be a useful bedside test, allowing for rapid diagnosis while sparing patients ionizing radiation and painful diagnostic procedures. Below we describe the sonographic steps to diagnose TAK, followed by three cases demonstrating the utility of ultrasound in identifying TAK.

**Image 1.**
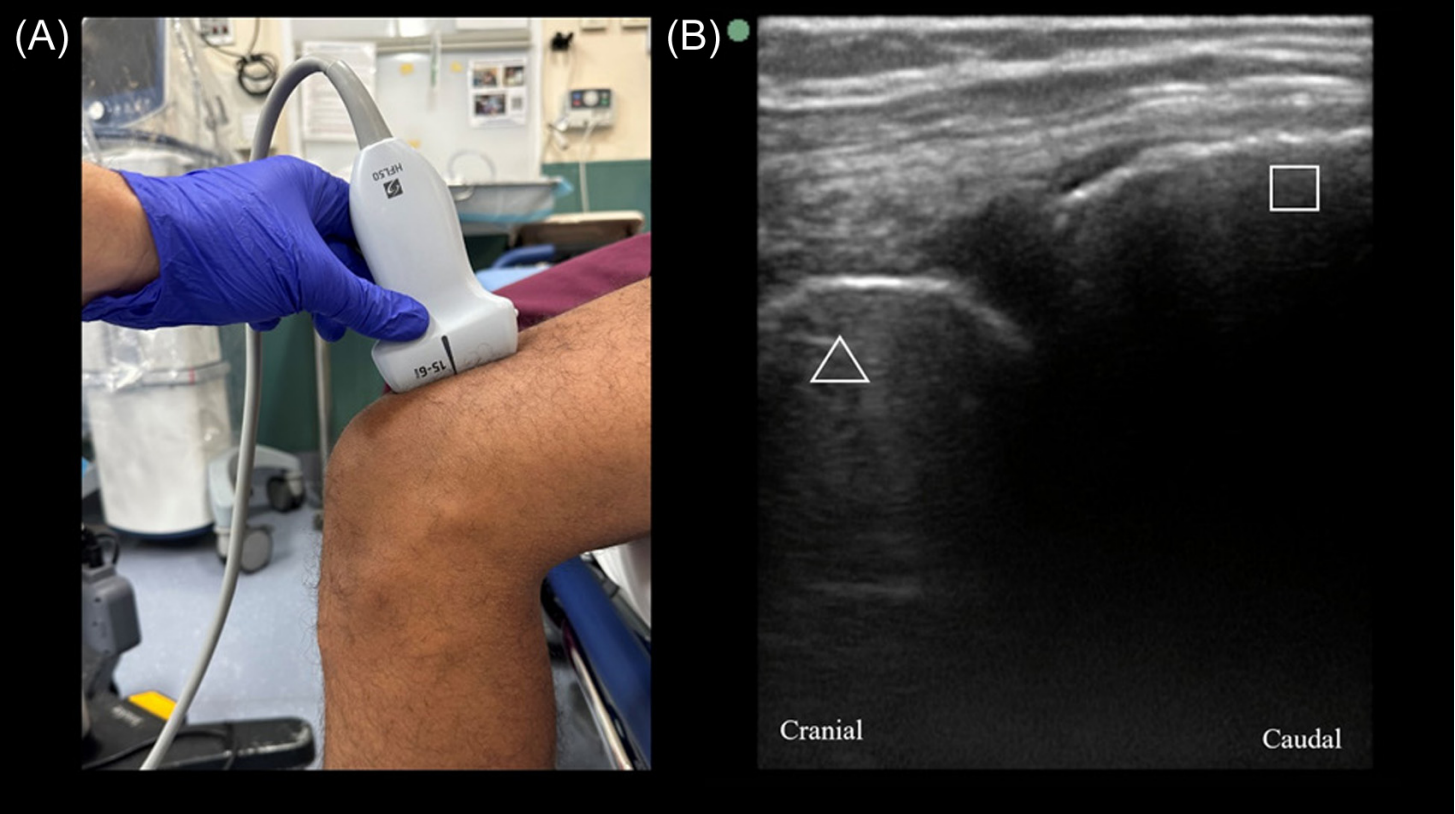
(A) The orientation of the probe placed in the sagittal plane in the suprapatellar region with the probe marker oriented cranially; (B) a scan showing a normal knee with the triangle indicating the distal femur and the square marking the patella.

Knee joints were scanned using a high-frequency linear probe in the sagittal plane, starting at the anterior knee in the suprapatellar region (Image 1A). Initial orientation of the probe collected views of the following structures in relation to the probe marker: patella (inferior aspect of the view); joint capsule; and distal femur (superior aspect of the view), as seen in Image 1B (normal view). Images captured include various views in the described orientation along a medio-lateral path. Abnormal findings were collected and are documented below.

## CASE SERIES

### Case 1

**Image 2.**
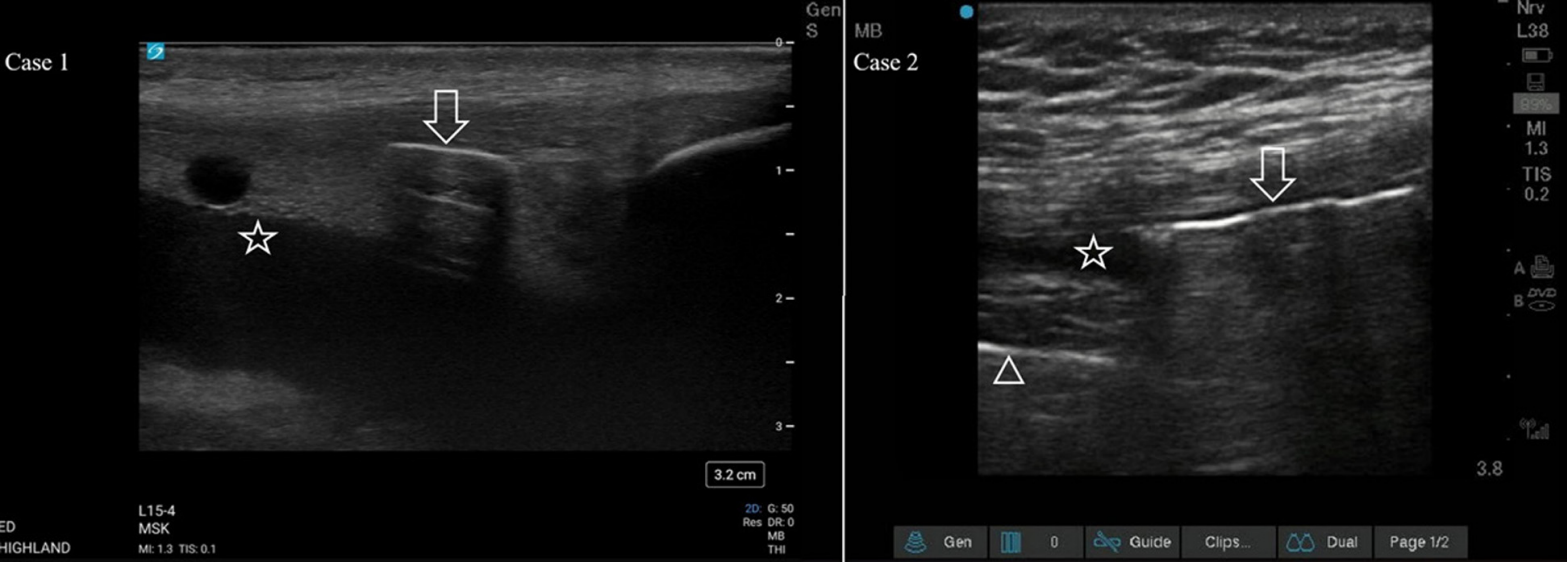
Case 1) A sagittal view of the suprapatellar recess showing a hyperechoic focus of air with posterior shadowing (arrow) within an effusion (star). The effusion has multiple components, which is indicative of lipohemarthrosis. Case 2) A sagittal view of the suprapatellar recess showing a hyperechoic focus of air with posterior shadowing and reverberation artifact (arrow). Note how the signal from the cortex of the femur (triangle) is obscured by the shadowing. A small joint effusion is also visualized (star).

A 20-year-old male presented to the ED with a gunshot wound to the anterior, right knee. He reported difficulty walking and limited range of motion of the knee. On physical examination, he had two missile wounds on the medial aspect of his leg: one superior to the patella and one inferior to the tibial plateau. The initial radiograph showed no occult fracture but demonstrated bullet fragments near the knee joint. A POCUS examination by the emergency physician (EP) scanning the suprapatellar recess showed internal hyperechoic linear structures concerning for free air (Image 2, Case 1) within hypoechoic effusion with anechoic bubbles concerning for lipohemarthrosis. The EP made the preliminary diagnosis of TAK. A CT was ordered to confirm TAK, but due to the overnight burden of trauma patients it was not performed until seven hours after presentation with interpretation taking an additional hour. Orthopedics took the patient to the operating room (OR) the following day for irrigation and debridement where TAK was confirmed. The patient was given a prophylactic seven-day course of cephalexin 500 milligrams (mg) every six hours and a one-week follow-up with orthopedics.

### Case 2

A 20-year-old male presented to the ED with a deep laceration to the left knee after a bicycle accident five hours prior. On examination, there was a deep, 15-centimeter (cm) long infrapatellar laceration with exposed but intact tendon. The patient was ambulatory, without neurologic deficits, and had full range of motion of the left knee. A POCUS examination of the left knee was performed by the EP and showed a hypoechoic joint effusion in the suprapatellar space that contained numerous mobile, hyperechoic structures with posterior shadowing along the superior aspect of the fluid collection, suggestive of TAK (Image 2, Case 2). Subsequent radiographs of the knee demonstrated no bony injury but a possible small focus of gas in the suprapatellar recess. Computed tomography confirmed the presence of gas within the knee joint with the additional finding of a small, left femoral condyle avulsion fracture. The wound was irrigated with normal saline and dressed. Orthopedics was consulted, and the patient was started on vancomycin intravenously. He was taken to the OR the next day for irrigation and primary repair of the joint capsule.

### Case 3

A 23-year-old female presented to the ED with a laceration near the right knee after a moderate-speed motor vehicle collision. On examination she had a 4-cm long laceration medial to the patella that extended into the subcutaneous tissue and significant pain with passive ranging of the knee. Radiographs of the knee did not demonstrate bony injury, but lucencies suspicious for air were noted by the EP in the region of the suprapatellar recess.

**Image 3.**
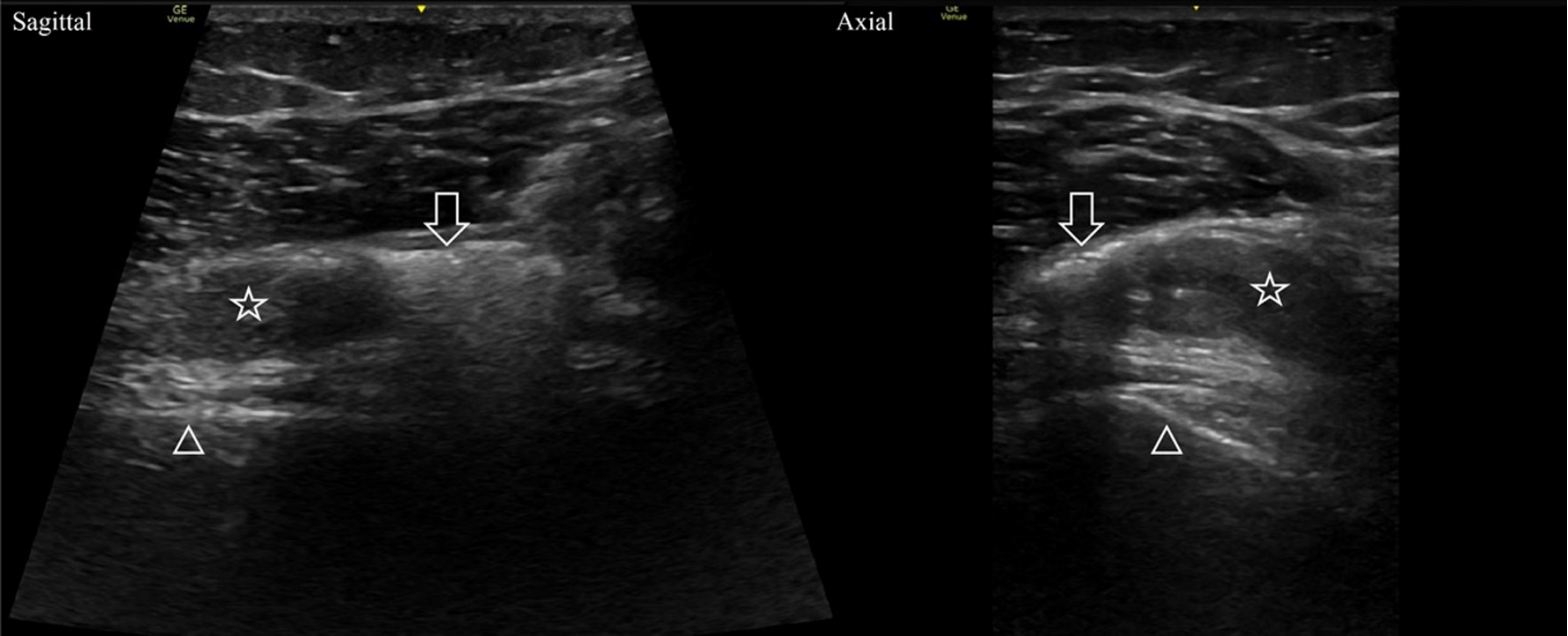
Sagittal and axial views of the distal femur showing hyperechoic foci with posterior acoustic shadowing (arrows) within a hypoechoic hemarthrosis (stars). Note the shadowing obscures the signal from the femur cortex (triangles).

A POCUS examination of the knee joint was performed by the EP showing a hypoechoic joint effusion in the suprapatellar recess with hyperechoic structures with posterior shadowing (Image 3). These findings were interpreted as a hemarthrosis with intra-articular air bubbles suspicious for TAK. Orthopedics was consulted, which confirmed the arthrotomy with a positive SLT. The patient was treated with one gram of cefazolin and tetanus vaccination update. Orthopedics performed saline irrigation of the joint and primary repair of the capsule laceration at the bedside. The patient was discharged on a prophylactic course of cephalexin 500 mg every six hours for five days. At two-week follow-up with orthopedics she had no signs of joint infection.

## DISCUSSION

Traumatic arthrotomy should be considered during the evaluation of a patient with periarticular wounds due to the risk of septic arthritis. Currently, the SLT is recommended to diagnose traumatic arthrotomy in cases where arthrotomy is not obvious on physical examination. Point-of-care ultrasonography is an alternative, non-invasive imaging modality that has been demonstrated to have utility in evaluating various knee pathologies. One meta-analysis found that ultrasound had a sensitivity of 85% and specificity of 93% for traumatic and atraumatic knee effusions compared to magnetic resonance imaging.^11^ Another prospective trial of patients with acute knee trauma found that ultrasonography was highly accurate compared to radiography in diagnosing intra-articular knee fractures when lipohemarthrosis was present.^12^ Point-of-care ultrasonography has the benefit of being free of ionizing radiation and lower cost than CT, with the drawback of being operator-dependent. Computed tomography is also a highly used resource, which may lead to delays in diagnosis such as in Case 1.

Here we describe three patients with proven TAK first detected by POCUS examinations. Prior to this writing, our literature review resulted in only one German-language case series where POCUS examinations diagnosed TAK in patients presenting with knee trauma and periarticular wounds.^13^ As in our series, the finding of intra-articular air led to the correct diagnosis. The acoustic impedance mismatch between air and soft tissue makes air highly echogenic with resultant posterior shadowing (Video). Reverberation artifacts may also be seen with larger air bubbles.^14^ Literature regarding the diagnostic performance of POCUS for diagnosing knee arthrotomy in live patients is lacking. One cadaver study found a sensitivity of 65% and specificity of 75% for 1 mL of intra-articular air.^15^ The diagnostic performance of POCUS for TAK should be further investigated.

## CONCLUSION

Point-of-care-ultrasonography has a wide array of applications in the evaluation of ED patients, including bone and joint pathology. This series suggests that POCUS may be effective in diagnosing traumatic arthrotomy of the knee, using intra-articular air as the positive finding. So far, the finding of intra-articular air in other modalities has been shown to be highly sensitive and specific for TAK. Additional studies should be undertaken to better elucidate the diagnostic performance of POCUS as a modality to diagnose TAK.

## Supplementary Information

Video. Intra-articular air is visualized as a hyperechoic line with posterior shadowing (white arrow) that obscures the underlying cortex signal (black arrow).

## References

[1] Colmer HG 4th Pirotte M Koyfman A et al . High risk and low prevalence diseases: traumatic arthrotomy. Am J Emerg Med. 2022;54:41–5. 35121477 10.1016/j.ajem.2022.01.013

[2] McKnight RR Ruffolo M Wally MK et al . Traumatic arthrotomies: Do they all need the operating room? J Orthop Trauma. 2021;35(11):612–8. 34387570 10.1097/BOT.0000000000002093

[3] Goldenberg DL . Septic arthritis. Lancet. 1998;351(9097):197–202. 9449882 10.1016/S0140-6736(97)09522-6

[4] Brubacher JW Grote CW Tilley MB . Traumatic arthrotomy. J Am Acad Orthop Surg. 2020;28(3):102–11. 31977606 10.5435/JAAOS-D-19-00153

[5] Browning BB Ventimiglia AV Dixit A et al . Does the saline load test still have a role in the orthopaedic world? A systematic review of the literature. Acta Orthop Traumatol Turc. 2016;50(6):597–600. 27979366 10.1016/j.aott.2016.01.004 PMC6197556

[6] Konda SR Howard D Davidovitch RI et al . The saline load test of the knee redefined. J Orthop Trauma. 2013;27(9):491–7. 23287768 10.1097/BOT.0b013e31828211f3

[7] Konda SR Davidovitch RI Egol KA . Computed tomography scan to detect traumatic arthrotomies and identify periarticular wounds not requiring surgical intervention: an improvement over the saline load test. J Orthop Trauma. 2013;27(9):498–504. 23287770 10.1097/BOT.0b013e31828219bc

[8] Konda SR Howard DO Gyftopoulos S et al . Computed tomography scan to detect intra-articular air in the knee joint: a cadaver study to define a low radiation dose imaging protocol. J Orthop Trauma. 2013;27(9):505–8. 23287769 10.1097/BOT.0b013e3182821505

[9] Perloff E Posner A Murtaza H et al . CT scan versus saline load test for detection of traumatic wrist arthrotomy. J Wrist Surg. 2021;11(2):154–60. 35478947 10.1055/s-0041-1735888 PMC9038302

[10] Konda SR Howard D Davidovitch RI et al . The role of computed tomography in the assessment of open periarticular fractures associated with deep knee wounds. J Orthop Trauma. 2013;27(9):509–14. 23412508 10.1097/BOT.0b013e31828b7001

[11] Meyer R Lin C Yenokyan G et al . Diagnostic utility of ultrasound versus physical examination in assessing knee effusions: a systematic review and meta-analysis. J Ultrasound Med. 2022;41(1):17–31. 33675099 10.1002/jum.15676

[12] Bonnefoy O Diris B Moinard M et al . Acute knee trauma: role of ultrasound. Clinical Imaging. 2007;31(2):147. 10.1007/s00330-006-0319-x 16786321

[13] Grechenig W Clement H Peicha G et al . Sonographischer Nachweis von Luft im Kniegelenk - eine experimentelle Studie und klinischer Fallbericht. Ultraschall Med. 2002;23(01):47–51. 11842372 10.1055/s-2002-20071

[14] Buttar S Cooper D Jr Olivieri P et al . Air and its sonographic appearance: understanding the artifacts. J Emerg Med. 2017;53(2):241–7. 28372830 10.1016/j.jemermed.2017.01.054

[15] Kongkatong M Thom C Moak J . Can ultrasound identify traumatic knee arthrotomy in a cadaveric model? J Emerg Med. 2019;57(3):362–6. 31375371 10.1016/j.jemermed.2019.06.012

